# How Astragalin Modulates Glucose Uptake and Insulin Secretion in β-Cell Lines

**DOI:** 10.3390/ph19030508

**Published:** 2026-03-20

**Authors:** Paola Miranda Sulis, Alice Lima Rosa Mendes, Paula Waiss Zanusso Bunick, Karina Cesca, Carine Royer, Bruna Antunes Zaniboni, Fernanda Carvalho Cavalari, Guilherme Brasil Pintarelli, André Luiz Andreotti Dagostin, Fátima Regina Mena Barreto Silva

**Affiliations:** 1 Instituto de Bioeletricidade Celular (IBIOCEL), Ciência & Saúde, Departamento de Bioquímica, Centro de Ciência Biológicas, Universidade Federal de Santa Catarina, Florianópolis 88037-000, Brazil; pasulis@hotmail.com (P.M.S.); alice.rosa@posgrad.ufsc.br (A.L.R.M.); waisspaula@gmail.com (P.W.Z.B.); cariroyer@unb.br (C.R.); brunaantunesz@outlook.com (B.A.Z.); fernanda.labenex@gmail.com (F.C.C.); guilherme.pintarelli@ufsc.br (G.B.P.); aladagostin@gmail.com (A.L.A.D.); 2 Departamento de Engenharia Química e de Alimentos, Centro de Tecnologia, Universidade Federal de Santa Catarina, Florianópolis 88037-000, Brazil; karinacesca@gmail.com; 3 Laboratório de Farmacologia Molecular, Universidade de Brasília, Brasília 70900-910, Brazil; 4 Faculdade de Ciências e Tecnologias em Saúde, Universidade de Brasília, Brasília 70900-910, Brazil; 5 Departamento de Engenharia de Controle, Automação e Computação, Universidade Federal de Santa Catarina, Blumenau 89065-200, Brazil; 6 Vollum Institute, Oregon Health and Science University, Portland, OR 97239, USA

**Keywords:** diabetes mellitus, medicinal plants, insulin secretion, patch-clamp, ionic channels

## Abstract

**Background/Objectives**: Type 2 diabetes mellitus (T2DM) is characterized by chronic hyperglycemia and insulin resistance, leading to progressive metabolic dysfunction. Flavonoids, such as astragalin, have reported antidiabetic potential; however, their direct effects on pancreatic β-cell ionic mechanisms and insulin secretion remain unclear. This study aimed to investigate the effects of astragalin on glucose uptake, insulin secretion, and membrane ionic currents in pancreatic β-cell lines. **Methods**: Murine MIN6 and rat INS-1 pancreatic β-cells were used as experimental models. Following astragalin treatment, glucose uptake was quantified by bioluminescence, and insulin secretion was measured by ELISA. Ionic currents were analyzed using the whole-cell patch-clamp technique. Selective pharmacological blockers targeting ATP-sensitive K^+^ channels (KATP), voltage-dependent K^+^ channels (K_v_), and L-type voltage-dependent Ca^2+^ channels were applied to elucidate the underlying mechanisms. **Results**: Astragalin increased glucose uptake in a time-dependent manner, reaching a plateau between 3 and 5 h. Insulin secretion was significantly enhanced after 1 h of exposure to 100 µM astragalin. Patch-clamp recordings demonstrated that astragalin reduced potassium channel currents in pancreatic β-cells. Pharmacological modulation confirmed the involvement of KATP, K_v_, and L-type Ca^2+^ channels. Verapamil attenuated the insulinotropic effect, supporting the role of calcium influx in astragalin-induced insulin exocytosis. **Conclusions**: Astragalin enhances glucose uptake and stimulates insulin secretion in pancreatic β-cells through modulation of potassium and calcium channels, promoting calcium-dependent exocytosis. These findings support its potential as a candidate for antidiabetic therapeutic strategies.

## 1. Introduction

Type 2 Diabetes Mellitus (T2DM) is a complex metabolic disorder, characterized by chronic hyperglycemia, resulting from impaired insulin secretion and/or insulin resistance, leading to dysregulation of carbohydrate, lipid, and protein metabolism. Persistent hyperglycemia promotes β-cell dysfunction, oxidative stress, mitochondrial impairment, and chronic low-grade inflammation, all of which contribute to progressive deterioration of glycemic control [1,2]. Increasing evidence indicates that T2DM involves a multifactorial immunometabolic dysregulation, including activation of inflammatory signaling pathways, cytokine production, oxidative stress, and alterations in intracellular calcium handling, which collectively exacerbate insulin resistance and β-cell dysfunction [3].

Natural bioactive compounds have attracted significant attention as potential modulators of these intracellular pathways. Among these, flavonoids (plant secondary metabolites that are widely distributed in the human diet) have been extensively investigated for their antidiabetic properties [4,5,6]. Experimental studies have demonstrated that flavonoid derivatives modulate glucose homeostasis through multiple mechanisms, including regulation of oxidative stress, activation of AMP-activated protein kinase (AMPK), modulation of insulin signaling pathways, and stimulation of insulin secretion [7]. However, most previous investigations have primarily focused on biochemical and molecular signaling pathways, with limited direct characterization of the electrophysiological modulation of ionic currents involved in β-cell stimulus–secretion coupling.

Astragalin (kaempferol-3-O-β-glucoside), a flavonol glycoside, has demonstrated multiple pharmacological activities, including anti-inflammatory, antioxidant, and antidiabetic effects [8,9,10]. Its antioxidant and anti-inflammatory properties have been associated with the modulation of oxidative stress and inflammatory signaling pathways implicated in metabolic disorders. In vivo studies in normoglycemic Wistar rats subjected to an oral glucose tolerance test (OGTT) demonstrated that astragalin (10 mg/kg) significantly reduced glycemia and increased plasma insulin levels following glucose overload [11]. Consistent with these findings, ex vivo experiments using isolated rat pancreatic islets showed that astragalin stimulates calcium influx and insulin secretion through mechanisms involving ATP-sensitive potassium (KATP) channels, L-type voltage-dependent calcium channels (L-VDCC), SERCA pump modulation, and activation of protein kinase C (PKC) and protein kinase A (PKA), as demonstrated using specific pharmacological modulators and ^45^Ca^2+^ as a functional tracer [11]. Despite these advances, the precise electrophysiological targets of astragalin and its direct modulatory effects on ionic currents remain incompletely defined.

To address this gap, the present study was designed to investigate both metabolic and electrophysiological mechanisms of astragalin in pancreatic β-cells using complementary experimental models selected according to their suitability. The murine MIN6 β-cell line was employed to evaluate glucose uptake and glucose-stimulated insulin secretion (GSIS), due to its preserved metabolic responsiveness to glucose and stable stimulus–secretion coupling machinery [12,13]. MIN6 cells are widely used for the assessment of β-cell metabolic function because they maintain reproducible insulin secretory responses under controlled experimental conditions. Although commonly described as a β-cell line, MIN6 cultures are not exclusively composed of pancreatic β-cells and may include other endocrine pancreatic cell populations. This is supported by reports demonstrating the detection of not only insulin secretion, but also somatostatin and glucagon release [14]. Owing to this characteristic, MIN6 cells partially recapitulate the cellular heterogeneity of pancreatic islets, representing a model that more closely resembles the physiological microenvironment and thus is suitable for insulin secretion assays.

For electrophysiological analyses, the rat INS-1 β-cell line was used due to its well-characterized ionic currents and high suitability for whole-cell patch-clamp recordings [15,16]. In contrast to MIN6 cells, INS-1 cultures consist predominantly of pancreatic β-cells, making them particularly appropriate for single-cell electrophysiological approaches in which it is essential to ensure that the recorded cell is specifically a β-cell. INS-1 cells have been extensively applied in studies investigating voltage-dependent calcium channels, ATP-sensitive potassium channels, and other ionic mechanisms regulating insulin secretion. Although derived from different species, both MIN6 and INS-1 cell lines preserve fundamental mechanisms of β-cell stimulus–secretion coupling. The combined use of these complementary models enabled the evaluation of glucose metabolism and insulin secretion in MIN6 cells and the direct electrophysiological characterization of ionic currents in INS-1 cells, providing a comprehensive mechanistic assessment of astragalin action in pancreatic β-cells.

## 2. Results

### 2.1. Glucose Uptake

**Figure 1:**
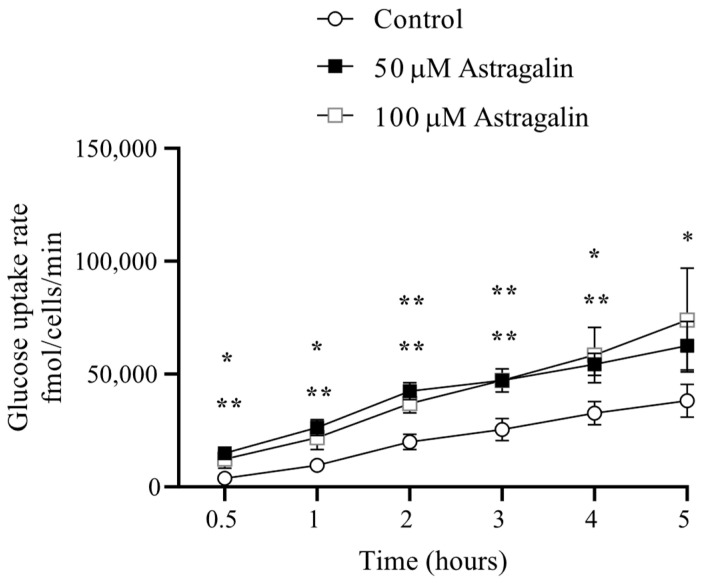
Effect of astragalin on glucose uptake in MIN6 cells. MIN6 cells were pretreated with astragalin (50 or 100 µM) for 30 min, followed by incubation with 2DG for 10 min. Intracellular 2DG accumulation was quantified using the Glucose Uptake-Glo™ assay, according to the manufacturer’s instructions and expressed as fmol/cell/min. Data are presented as means ± SEM of three independent experiments (*n* = 3). Statistical analyses were performed using one-way ANOVA followed by Tukey’s post hoc test. * *p* ≤ 0.05; ** *p* ≤ 0.01 vs. control.

Astragalin significantly increased glucose uptake in MIN6 cells following a 30 min pretreatment and a defined 10 min exposure to 2-deoxyglucose (2DG). Intracellular 2-deoxyglucose accumulation, quantified using the Glucose Uptake-Glo™ assay, was significantly higher in astragalin-treated cells, compared to control conditions (Figure 1). Both concentrations tested (50 and 100 µM) promoted enhanced glucose uptake, with 100 µM showing a greater effect. When normalized to cell number and expressed as fmol/cell/min, these findings indicate that astragalin enhances glucose uptake capacity in MIN6 cells under the defined experimental conditions.

### 2.2. Insulin Secretion

**Figure 2:**
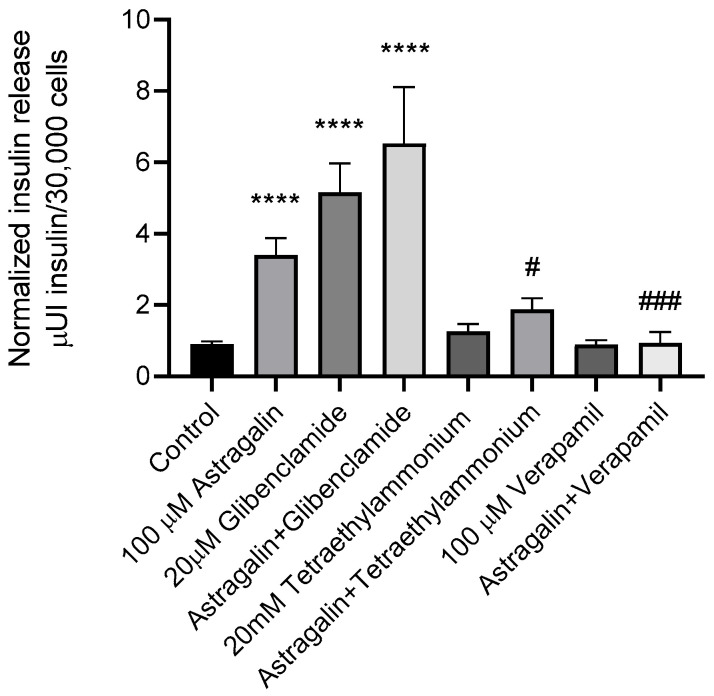
Effect of glibenclamide, TEA, and verapamil on astragalin-induced insulin secretion in MIN6 cells. MIN6 cells were incubated for 1 h under the indicated treatment conditions. Insulin secretion was quantified using ELISA and expressed as μUI insulin/30,000 cells. Data are presented as means ± SEM of two independent experiments (*n* = 4 per group). Statistical analyses were performed using one-way ANOVA followed by Tukey’s post hoc test. **** *p* ≤ 0.001 vs. control; # *p* ≤ 0.05 and ### *p* ≤ 0.001 vs. astragalin group.

In the insulin secretion assay, treatment with astragalin (100 µM, 1 h) significantly increased secretion relative to the control, with an effect similar to that observed with glibenclamide (Figure 2). Notably, the combination of astragalin and glibenclamide elicited an even greater insulin secretion than either compound alone.

In groups treated with TEA, either alone or in combination with astragalin, a significant reduction in insulin secretion was observed, compared to astragalin alone. Similarly, treatment with verapamil, either alone or combined with astragalin, reduced insulin secretion and attenuated the stimulatory effect induced by astragalin.

### 2.3. Electrophysiology (Patch Clamp) Results

**Figure 3:**
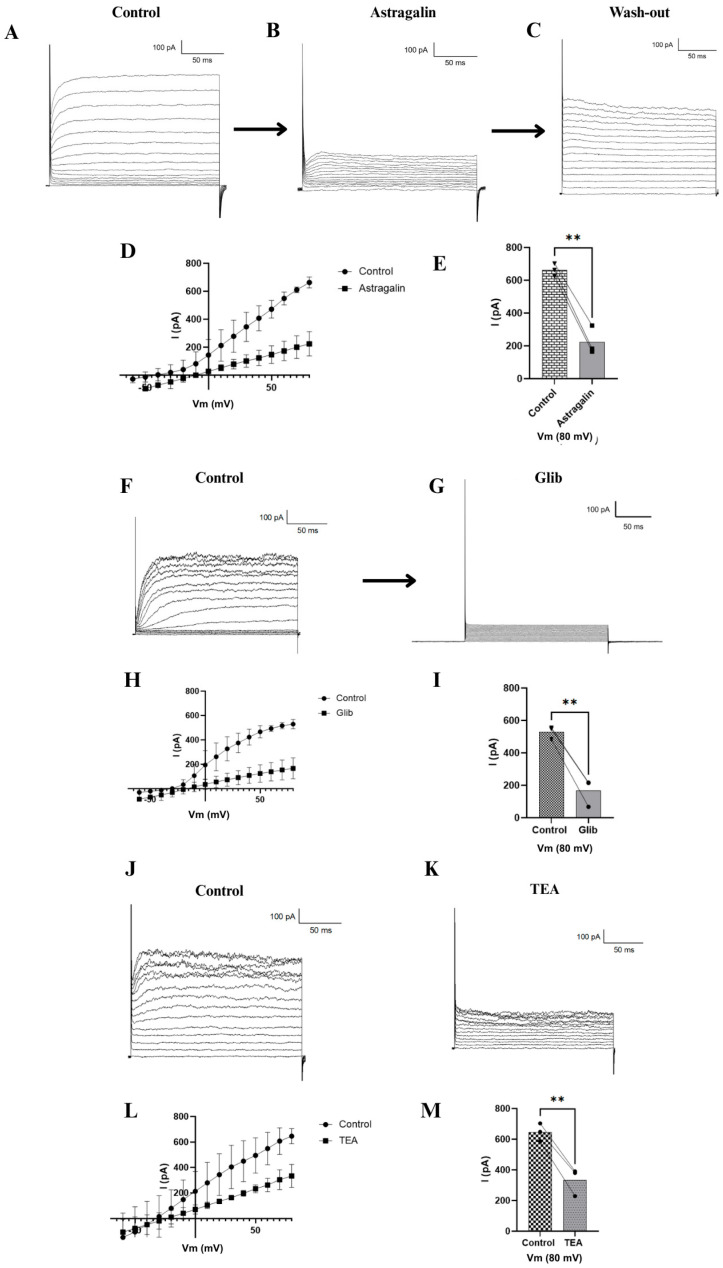
Results from electrophysiological assays using INS-1 cells. Control currents related to astragalin are shown in (**A**), effects of astragalin on K^+^ currents (**B**), and followed by wash out K^+^ currents (**C**), representative I–Vm relationship (control vs astragalin) is presented in (**D**), statistical analyses (control vs astragalin) are shown in (**E**)**.** Control currents related to glibenclamide group are shown in (**F**), the effects of glibenclamide on K^+^ currents (**G**), representative I–Vm relationship (control vs glib) is presented in (**H**), statistical analyses (control vs glib) are shown in (**I**). Control currents related to TEA group are shown in (**J**), and TEA effects on K^+^ currents (**K**), representative I–Vm relationship (control vs TEA) is presented in (**L**), statistical analyses (control vs TEA) are shown in (**M**). Data expressed as mean ± standard error of the mean (SEM), and *n* = 3 for each tested compound. Statistically significant differences were indicated by ** *p* ≤ 0.01.

Figure 3A shows that once stable access to the cell was achieved via membrane rupture with the micropipette, it was possible to record potassium channel activity, as demonstrated by an electrical current of approximately 790 pA. Upon perfusion with 100 µM astragalin, a progressive reduction in current amplitude was observed. The inhibition became detectable approximately 5 min after drug application, with a marked increase between 25 and 30 min, as illustrated in the representative trace. At this time point, steady-state current amplitude decreased to approximately 200 pA (Figure 3B). Time-matched vehicle controls were performed, and no significant current rundown was observed over the recording period. Drug-free extracellular solution was perfused again, leading to a partial recovery of the amplitude (Figure 3C). To confirm the modulation of potassium channels, two specific inhibitors were tested. Glibenclamide reduced the current to approximately 100 pA (Figure 3G), while tetraethylammonium (TEA) reduced it to around 400 pA (Figure 3K). Representative control recordings for these inhibitors are shown in Figure 3F,J.

The current–voltage (I–Vm) relationships presented in Figure 3D,H,L demonstrate that astragalin, glibenclamide, and TEA all decreased potassium channel conductance, compared to the control condition. Statistical analysis performed using one-way ANOVA (*n* = 3) confirmed that all tested compounds significantly reduced the current (Figure 3E,I,M). Among them, astragalin and glibenclamide exhibited the most pronounced inhibitory effects, followed by TEA.

## 3. Discussion

The increase in intracellular 2DG accumulation observed in MIN6 cells following astragalin administration indicates that this flavonoid enhances glucose uptake under the experimental conditions defined. In pancreatic β-cells, glucose uptake is primarily mediated by GLUT2 transporters and is tightly coupled to glycolytic flux and mitochondrial oxidative phosphorylation, which together regulate intracellular ATP production [17,18]. Elevated ATP levels promote closure of ATP-sensitive potassium (KATP) channels, leading to membrane depolarization, opening of voltage-dependent calcium channels (VDCC), calcium influx, and insulin exocytosis [19,20]. Therefore, enhanced glucose uptake may directly contribute to amplification of the stimulus–secretion coupling cascade.

In addition to its effects on ionic mechanisms, astragalin has been reported to modulate intracellular metabolic signaling pathways. Rey et al. [11] demonstrated that astragalin enhances calcium influx and insulin secretion in rat pancreatic islets via mechanisms involving KATP channels, L-type calcium channels, and activation of PKA and PKC pathways. Furthermore, Sun et al. [21] reported that astragalin activates the AMPK–PGC-1α axis, stimulates mitochondrial biogenesis, and reduces oxidative stress in metabolic disease models. AMPK functions as a cellular energy sensor and regulates mitochondrial dynamics and metabolic efficiency, while PGC-1α is a master regulator of mitochondrial biogenesis and oxidative metabolism [22,23]. Activation of these pathways may improve mitochondrial function and ATP generation, thereby reinforcing β-cell metabolic responsiveness.

Taken together, these findings suggest that astragalin may enhance β-cell function through coordinated modulation of glucose transport, mitochondrial bioenergetics, and calcium-dependent signaling pathways. Such multifaceted regulation supports the concept that astragalin acts as both a metabolic and electrophysiological modulator of pancreatic β-cells.

The secretion of insulin in pancreatic β-cells, such as MIN6 cells, depends on a precise coupling between glucose metabolism, membrane electrical activity, and ionic fluxes, particularly K^+^ and Ca^2+^. K^+^-ATP channels constitute the central link between metabolic status and membrane potential, whereas voltage-dependent K^+^ channels (K_v_) and L-type voltage-dependent Ca^2+^ channels (L-VDCCs) refine the duration of the action potential and the pattern of Ca^2+^ entry, ultimately determining insulin granule exocytosis [19,20,24,25]. In this context, our findings indicate that astragalin acts as a potent secretagogue, integrating into multiple stages of the stimulus–secretion coupling cascade.

The effects of astragalin and glibenclamide (a classic sulfonylurea that blocks K^+^-ATP channels and triggers membrane depolarization, followed by the opening of L-VDCC) [20,25] indicate that astragalin does not simply compete for the same binding site as glibenclamide on the K^+^-ATP channel, but likely acts through complementary mechanisms, such as modulating additional ionic channels or components of the exocytotic machinery, in alignment with the multifaceted role of flavonoids in the electrophysiological modulation of excitable cells [26].

The involvement of K_v_ channels in the action of astragalin is supported by the reduction in insulin secretion observed in the presence of TEA. Considering that TEA is a classical blocker of K_v_ channels, the results suggest that the secretagogue effect of astragalin depends, at least in part, on the functional integrity of K_v_ channels. Previous studies have demonstrated that inhibition of K_v2.1_ prolongs depolarization, increases Ca^2+^ influx, and enhances insulin secretion [27]. Moreover, recent evidence indicates that K_v_ channels such as KCNH6 can directly regulate exocytosis through interactions with secretory machinery proteins, including Munc18-1, independently of K^+^ conductance [28].

The result obtained with verapamil further reinforces the critical role of Ca^2+^ in the action of astragalin. Since verapamil blocks L-VDCC, these findings indicate that the secretagogue action of astragalin depends on Ca^2+^ entry through these channels. This interpretation is supported by studies demonstrating that Ca_V1.2_ channels are strongly coupled to insulin secretion and that alterations in their expression impair the secretory response [29,30].

In addition, reports in the literature show that chronic inhibition of K^+^-ATP channels, either due to loss-of-function mutations or prolonged exposure to sulfonylureas, can reduce Ca^2+^ sensitivity and lead to β-cell exhaustion [24,31]. In this context, astragalin modulates multiple pathways, promoting depolarization via K^+^-ATP channels (in synergy with glibenclamide), thereby prolonging depolarizing events and influencing exocytosis through K_v_ channels, and amplifying Ca^2+^ influx through L-type channels. This coordinated action suggests a potentially advantageous pharmacological profile that combines a strong secretagogue effect with distributed action across the stimulus–secretion cascade.

The electrophysiology data presented indicate that astragalin exerts its effects through the modulation of potassium channels. The partial recovery of the current after washout further supports a direct and reversible modulatory effect on these channels. Both ATP-sensitive and voltage-dependent potassium channels are involved in the insulin secretion process in pancreatic β-cells. Closure of ATP-sensitive potassium channels leads to membrane depolarization, which in turn triggers the opening of calcium channels and subsequent insulin release [32]. Additionally, inhibition of voltage-dependent potassium channels prevents membrane repolarization, prolonging the depolarized state of the cell and thereby sustaining insulin secretion [33]. These findings are consistent with those of a study by Trezza et al. [34], who used patch-clamp experiments in rat arterial myocytes to demonstrate that quercetin, the aglycone of astragalin, is also capable of inhibiting K^+^-ATP channels.

Taken together, these findings provide evidence that astragalin modulates potassium channel activity and directly influences glucose homeostasis in pancreatic β-cells, highlighting its potential as a metabolic modulator and a promising candidate for future studies aimed at developing novel β-cell-targeted antidiabetic therapies.

Beyond its mechanistic findings, this study has potential clinical implications. The ability of astragalin to enhance glucose uptake and potentiate insulin secretion through the modulation of K^+^ and Ca^2+^ channels suggests a pharmacological profile that may complement existing antidiabetic therapies. In particular, its synergistic interaction with glibenclamide indicates that astragalin could enhance β-cell responsiveness while acting through partially distinct mechanisms. Such multi-target modulation aligns with the current therapeutic trend toward agents capable of acting on multiple components of glucose homeostasis [35].

Several translational challenges must be considered, however. First, our findings are based on in vitro β-cell models, which do not fully recapitulate the systemic metabolic complexity of T2DM. Second, flavonoids such as astragalin often exhibit limited bioavailability due to rapid metabolism, poor intestinal absorption, and extensive biotransformation, which may restrict their therapeutic efficacy in vivo [36]. Additionally, long-term modulation of ion channels in β-cells must be carefully evaluated to avoid potential risks of β-cell exhaustion, a phenomenon observed with chronic overstimulation of insulin secretion [37].

As such, although the current findings support astragalin as a promising metabolic modulator, further in vivo studies, pharmacokinetic characterization, and long-term safety assessments are necessary before considering clinical translation.

## 4. Materials and Methods

### 4.1. Chemical Compounds

Astragalin, glibenclamide and verapamil were purchased from Sigma-Aldrich (St. Louis, MO, USA), while tetraethylammonium (TEA) was obtained from Alfa Aesar (Ward Hill, MA, USA). Dimethyl sulfoxide (DMSO) was used as the vehicle for astragalin and glibenclamide stock solutions, whereas TEA and verapamil were diluted in ultrapure water. The final concentration of DMSO in all experimental conditions did not exceed 0.1% (*v*/*v*). Equivalent vehicle controls were included in all assays. This concentration has been widely reported not to significantly affect cell viability, membrane integrity, or cellular metabolic activity in in vitro models [38,39].

### 4.2. Cell Culture

MIN6 cells (code 0263), pancreatic tumor cells derived from Mus musculus, were obtained from the Rio de Janeiro Cell Bank (Rio de Janeiro, RJ, Brazil). Cells were used at passage 48 for glucose uptake and insulin secretion assays. Cells were cultured in low-glucose Dulbecco’s Modified Eagle Medium (DMEM) supplemented with 2 mM L-glutamine, 10% fetal bovine serum (FBS), 100 U/mL penicillin, and 100 μg/mL streptomycin, under standard conditions (37 °C, 5% CO_2_). Cultures were maintained according to the Cell Bank guidelines (https://bcrj.org.br, accessed on 17 March 2026), with passages performed at ~80% confluence. For subsequent experiments, cells in the exponential growth phase (log) were seeded at 50–80% confluence [14].

INS-1 cells are a rat pancreatic β-cell line derived from an insulinoma, widely used as an in vitro model to study insulin secretion, ion channel activity, and β-cell metabolism [40]. Cells were obtained from Merck S.A (Polvilho, São Paulo, Brazil), and used at passage 4 for electrophysiological recordings. The cells were cultured in RPMI 1640 medium supplemented with 10% heat-inactivated FBS, 1 mM sodium pyruvate, 10 mM HEPES, 50 µM β-mercaptoethanol, and antibiotics (100 U/mL penicillin and 100 µg/mL streptomycin), and maintained at 37 °C in a humidified atmosphere with 5% CO_2_. This model provides a robust and reproducible system for investigating pancreatic β-cell physiology and pharmacology.

### 4.3. Glucose Uptake Assay

The glucose uptake assay was performed in MIN6 cells using the bioluminescent Glucose Uptake-Glo™ Assay (Promega, São Paulo, Brazil), based on the detection of 2-deoxyglucose (2DG). For glucose uptake experiments, MIN6 cells were pretreated with astragalin (50 or 100 µM) for 30 min at 37 °C in low-glucose medium, washed twice with PBS, and treated with astragalin (50 or 100 µM), saline solution (negative control), or 2DG6P (positive control). After 30 min, cells were washed again, incubated with 2-deoxyglucose (2DG) for 10 min at 37 °C, and subsequently treated with Stop Buffer, Neutralization Buffer, and the detection reagent. The reaction was incubated for 30 min at 25 °C, followed by luminescence measurement in a microplate reader after an additional 5 h. Results were expressed as fmol/cell/min (*n* = 3 for each group), according to the manufacturer’s instructions [41]. Statistical analysis was carried out using one-way ANOVA for multiple comparisons. Statistical significance was considered at *p* ≤ 0.05.

### 4.4. Insulin Measurement

The insulin concentration was measured using an enzyme-linked immunosorbent assay (ELISA) kit for insulin from Thermo Scientific (Waltham, MA, USA), based on the sandwich principle. In this method, microtiter plate wells are coated with a monoclonal antibody specific to a unique antigenic site on the insulin molecule. For this purpose, 30,000 MIN-6 cells were added to the insulin ELISA kit plate, according to the datasheet instructions. The cells were then incubated for 1 h in Krebs-Hepes buffer containing 5 mM glucose, in the presence and absence of astragalin with activators/inhibitors of ion channels of interest for insulin secretion. Subsequently, a test sample aliquot containing endogenous insulin was incubated in the coated well, where binding occurred with an enzyme conjugate consisting of a biotin-conjugated anti-insulin antibody. After incubation, the unbound conjugate was removed by washing. During the second incubation step, the streptavidin–peroxidase enzyme complex binds to the biotin–anti-insulin antibody. The amount of complex formed is directly proportional to the insulin concentration present in the sample. Upon addition to the substrate solution, the intensity of the developed color reflects the insulin concentration in the sample. Data were expressed as insulin content: μUI insulin/30,000 cells [42]. Statistical analysis was carried out using one-way ANOVA for multiple comparisons. Statistical significance was considered at *p* ≤ 0.05.

### 4.5. Patch-Clamp Technique

Whole-cell patch clamp electrophysiological recordings were performed on INS-1 cells (rat insulinoma pancreatic β-cells) using a MultiClamp 700B amplifier (Molecular Devices Corp., San Jose, CA, USA) connected to a Digidata 1440A digitizer and controlled by the pCLAMP 10 software (Molecular Devices Corp., San Jose, CA, USA). Given the electrophysiological profile of pancreatic β-cells, which express both ATP-sensitive potassium (KATP) channels and voltage-dependent potassium (K_v_) channels, the experimental conditions were designed to allow for the recording of total outward potassium currents without prior selective blockade. This approach was designed to primarily demonstrate whether astragalin is capable of inhibiting total potassium currents in pancreatic β-cells. Cells were cultured on coverslips pre-treated with poly-L-lysine to enhance adhesion and visualized under an Olympus IX81 inverted microscope integrated into the patch clamp setup. The extracellular solution used during recordings contained: 115 mM NaCl, 5 mM KCl, 1 mM MgCl_2_, 1 mM CaCl_2_, 3 mM glucose, and 10 mM HEPES (280 mOsm/kg H_2_O, pH 7.4). The internal pipette solution, optimized for potassium channel recordings, consisted of 127 mM KCl, 1 mM CaCl_2_, 1 mM MgCl_2_, 10 mM EGTA and 10 mM HEPES (290 mOsm/kg H_2_O, pH 7.4). The osmolality of solutions was carefully monitored using a VAPRO^®^ 5600 vapor pressure osmometer (Wescor Inc., Logan, UT, USA), ensuring that astragalin addition did not alter osmotic conditions. Patch pipettes were fabricated from borosilicate glass capillaries (Sutter, 1.5 × 0.86 mm × 10 cm) using a Micropipette Puller P-1000 (Sutter Instrument Company, Novato, CA, USA). Membrane currents were recorded in response to voltage steps ranging from −60 mV to +80 mV in 10 mV increments. Pipette resistance ranged between 6 and 8 MΩ. Pharmacological assays were conducted by perfusing the cells with glibenclamide (20 nM), TEA (20 mM), or astragalin (100 µM) [42]. Data analysis was performed using Clampfit 11.4.1 (Molecular Devices Corp., Sunnyvale, CA, USA) and Origin 2025b (OriginLab Corporation, One Roundhouse Plaza, Northampton, MA, USA). Experiments were conducted with *n* = 4, and statistical analysis was carried out using one-way ANOVA for multiple comparisons. Statistical significance was considered at *p* ≤ 0.05.

## 5. Conclusions

In summary, the results of this study demonstrate that astragalin enhances both glucose uptake and insulin secretion in pancreatic β-cells, involving multiple ionic channels and calcium- and AMPK-dependent signaling pathways. The modulation of ATP-sensitive K^+^ channels, voltage-dependent K^+^ channels, and L-type Ca^2+^ channels highlight the multifaceted role of this flavonoid in stimulus–secretion coupling. These findings underscore the potential of astragalin as a metabolic modulator and secretagogue, supporting its relevance as a candidate for novel therapeutic strategies in the management of T2DM.

## Data Availability

The original contributions presented in this study are included in the article. Further inquiries can be directed to the corresponding author.

## Abbreviations

The following abbreviations are used in this manuscript:

AMPK	AMP-Activated Protein Kinase
ANOVA	Analysis of Variance
ATP	Adenosine Triphosphate
Ca_V1.2_	Voltage-Gated Calcium Channel Subtype 1.2
DMEM	Dulbecco’s Modified Eagle Medium
DMSO	Dimethyl Sulfoxide
ELISA	Enzyme-Linked Immunosorbent Assay
FBS	Fetal Bovine Serum
GLUT2	Glucose Transporter Type 2
K^+^-ATP	ATP-Sensitive Potassium Channels
K_v_	Voltage-Gated Potassium Channels
PKA	Protein Kinase A
PKC	Protein Kinase C
RPMI	Roswell Park Memorial Institute Medium
SERCA	Sarco/Endoplasmic Reticulum Ca^2+^-ATPase
T2DM	Type 2 Diabetes Mellitus
TEA	Tetraethylammonium
VDCC	Voltage-Dependent Calcium Channels
2DG	2-Deoxyglucose
2DG6P	2-Deoxyglucose-6-Phosphate
2-NBDG	2-(N-(7-Nitrobenz-2-oxa-1,3-diazol-4-yl)Amino)-2-Deoxyglucose
